# DIRS and Ngaro Retrotransposons in Fungi

**DOI:** 10.1371/journal.pone.0076319

**Published:** 2013-09-25

**Authors:** Anna Muszewska, Kamil Steczkiewicz, Krzysztof Ginalski

**Affiliations:** Laboratory of Bioinformatics and Systems Biology, CeNT, University of Warsaw, Warsaw, Poland; University of Ottawa, Canada

## Abstract

Retrotransposons with a tyrosine recombinase (YR) have been discovered recently and lack thorough annotation in fungi. YR retrotransposons are divided into 3 groups: DIRS, Ngaro and VIPER (known only from kinetoplastida). We used comparative genomics to investigate the evolutionary patterns of retrotransposons in the fungal kingdom. The identification of both functional and remnant elements provides a unique view on both recent and past transposition activity. Our searches covering a wide range of fungal genomes allowed us to identify 2241 YR retrotransposons. Based on CLANS clustering of concatenated sequences of the reverse transcriptase (RT), RNase H (RH), DNA N-6-adenine-methyltransferase (MT) and YR protein domains we propose a revised classification of YR elements expanded by two new categories of Ngaro elements. A phylogenetic analysis of 477 representatives supports this observation and additionally demonstrates that DIRS and Ngaro abundance changed independently in Basidiomycota and Blastocladiomycota/Mucoromycotina/Kixellomycotina. Interestingly, a single remnant Ngaro element could be identified in an Ascomycota genome. Our analysis revealed also that 3 Pucciniomycotina taxa, known for their overall mobile element abundance and big genome size, encode an elevated number of Ngaro retrotransposons. Considering the presence of DIRS elements in all analyzed *Mucoromycotina* , Kickxellomycotina and Blastocladiomycota genomes one might assume a common origin of fungal DIRS retrotransposons with a loss in Dicarya. Ngaro elements described to date from Opisthokonta, seem to have invaded the common ancestor of Agaricomycotina and Pucciniomycotina after Ustilagomycotina divergence. Yet, most of analyzed genomes are devoid of YR elements and most identified retrotransposons are incomplete.

## Introduction

Transposable elements are one of the key genome architects [1] shaping genomes *via* chromosomal rearrangements, creating new gene neighborhoods [2], and altering gene expression [3]. Their amplification is related to speciation and differentiation [4].

Genomes developed multiple mechanisms to prevent the expansion of transposable elements. At least four of the main transposon silencing mechanisms have been described from fungi: repeat-induced point mutation (RIP), methylation, quelling and sex-induced silencing (SIS) [5-8]. Nevertheless, some fungal genomes, for example the genomes of *Puccinia* *graminis* f. sp. *tritic* and *Melampsoralaricis* - *Populina* , have expanded at least twofold compared to other Basidiomycota [9].

Transposons are traditionally divided into Class I (retroelements) and Class II (DNA transposons) elements based on their mobility mechanism. Class I elements (retrotransposons) are characterized by utilizing an RNA intermediate in their transposition cycle. Retrotransposons encode a reverse transcriptase (RT) homologous to retroviral RTs to synthesize a cDNA copy using an RNA as a template. Contrarily, Class II transposons function in an excision and insertion fashion. Their basic architecture is simpler, similar to bacterial insertion sequences (IS) with a single transposase open reading frame. Yet, also more complex DNA transposon types are already known: helitrons and mavericks [10,11].

Tyrosine recombinase (YR)-encoding elements belong to Class I retrotransposons. However, their integration mechanism is distinct from the one observed for other retroelements (LTR retrotransposons, non-LTR retrotransposons and Penelope elements), in which the nuclease activity necessary for integration is performed by a DDE integrase. Since the mechanism of integrase activity must be correlated with the structure of a mobile element, YR-encoding elements display also a different organization of its components. YR retrotransposons are divided into 3 groups [12]: DIRS, Ngaro and VIPER (currently known only from Kinetoplastida [13]). Here, we focus on Ngaro and DIRS retrotransposons that could be identified in fungal genomes.

**Figure 1:**
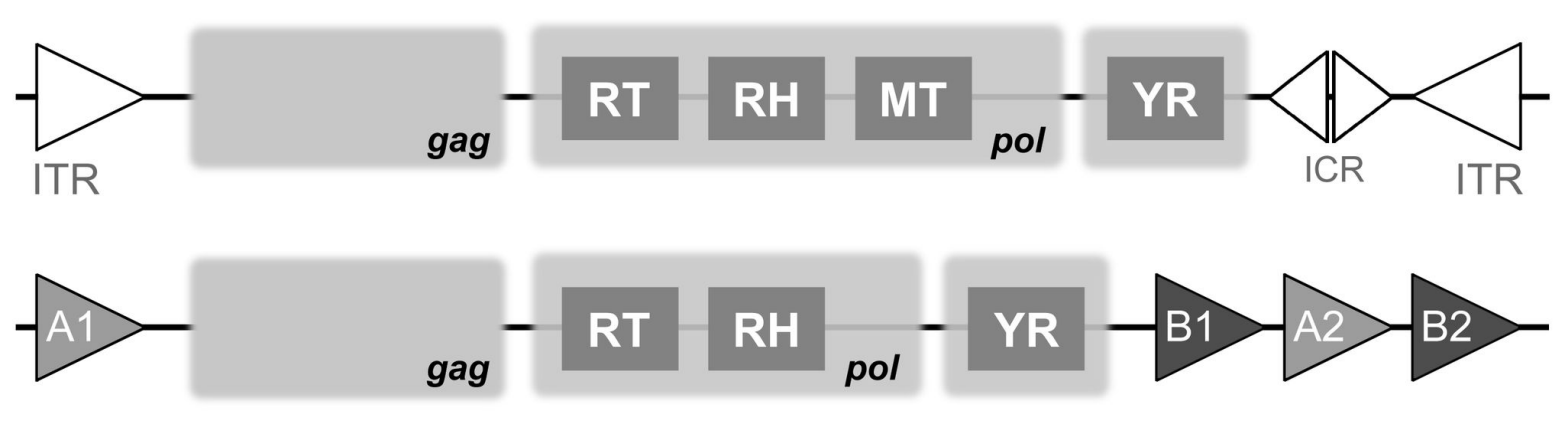
A schematic representation of DIRS and Ngaro architectures. Abbreviations: YR, tyrosine recombinase; RT, reverse transcriptase; RH, RNase H; MT, DNA N-6-adenine-methyltransferase; ICR, internal complementary region; ITR, inverted terminal repeats; A1-B1-A2-B2 repeat pairs.

YR-encoding elements consist of central *gag, pol* and tyrosine recombinase (YR) open reading frames (ORFs) flanked with terminal repeats (Figure 1). The *pol* ORF includes a reverse transcriptase (RT), a RNase H (RH) and, in case of DIRS, a domain similar to bacterial and phage DNA N-6-adenine-methyltransferase (MT) [14]. Compared to the retroviral *pol*, both aspartic protease and DDE integrase are absent from YR retrotransposons [15]. The YR ORF can be frameshifted and overlap with other ORFs [15]. The RT (RNA polymerase) catalyzes RNA-templated synthesis of cDNA, while RNase H is responsible for the degradation of the RNA template from the synthesized DNA-RNA hybrid. Finally, YR integrase inserts the retrotransposon cDNA into the genome of a host cell. The boundaries of DIRS and Ngaro elements are defined by terminal repeats. DIRS retrotransposons have inverted terminal repeats (ITRs), while Ngaro repeats are arranged in A-*pol*-B-A-B order [15].

DIRS retrotransposons are broadly distributed among Unikonta, however are absent from mammals genomes [14]. The first fungal DIRS was identified in *Phycomycesblakesleeanus* (Mucoromycotina) by Ruiz-Perez et al. in 1996 [16] but its proper classification was proposed a few years later by Goodwin and Poulter [15]. Up to date, fungal DIRS elements have been reported solely within early branching fungal taxa: *Rhizopus oryzae* (Mucoromycotina) and *Allomycesmacrogynus* (Blastocladiomycota) [14]. Ngaro elements are poorly described in the literature and all known representatives were identified only in Opisthokonta including *Coprinopsis* *cinerea* and *Phanerochaete chrysosporium* genomes [15]. Additionally, according to RepBase Reports [17], Ngaro are abundant and diverse in some of the Basidiomycota genomes ( *Melampsoralaricis-populina* , *Puccinia* *graminis* ). Consequently, most of the Ngaro consensus sequences stored in RepBase come from the aforementioned genomes.

In general, little is known about YR retrotransposon distribution and abundance in fungal genomes compared to the amount of data on LTR retrotransposons. There has been no whole kingdom analysis of YR retrotransposons in fungi, yet. Here we present the results of large-scale searches for YR TEs in 177 fungal genomes. We looked at both full-length and truncated elements to investigate recent and ancient transposition activities. These analyses enabled us to hypothesize about DIRS and Ngaro ancestry and their evolutionary history in fungi. Our results point at the separate evolution of these two dominant groups of YR retrotransposons in the fungal kingdom. These data corroborate the previous observations that transposon expansions in fungi usually involve an increase both in copy number of individual elements per family and in the number of YR retrotransposon families.

## Materials and Methods

### Genomes

Genome sequences were obtained from sequencing consortia: Fungal Genome Initiative (BROAD Institute; http://www.broadinstitute.org/) and the DOE Joint Genome Institute (JGI; http://www.jgi.doe.gov/). All downloads were performed before November 4^th^, 2011 respecting the 12 months memorandum. A list of genomes used in this study is available in File S1. Genomic context analysis was performed using the publicly available data in BROAD genome browser at *Aspergillus* Comparative Database.

### YR retrotransposon detection

To detect all available sequences corresponding to DIRS and Ngaro retrotransposons we used RepeatModeler followed by RepeatMasker 3.3.0 [18]. RepeatModeler was run separately on each genome in order to obtain consensus sequences for genome-specific retrotransposon classes. All genomic consensus sequences together with the whole RepBase library of manually curated mobile elements and repetitive DNA sequences [19] were used as the reference library for RepeatMasker searches. Only elements encoding at least one of the key four proteins: RT, RNase H, YR and MT were used for further analyses. Ugene toolkit was used to find repeats in YR retrotransposon sequences [20].

### YR retrotransposon protein domain identification

Since RT, RNase H, YR and MT protein domains are key elements for YR retrotransposon classification, identified repetitive elements were screened for these retrotransposon-related protein domains. Each putative YR retrotransposon was translated into 6 frames with transeq from the EMBOSS package [21] and scanned with HMMsearch (from HMMer3.0 package [22]) against Pfam HMM (Hidden Markov Model) profiles (using pfam_scan.pl with E-value threshold 0.01) and RPS-BLAST against CDD profiles (with E-value threshold 0.001). All profiles corresponded to 4 protein domains catalogued in Pfam26 [23] and CDD databases [24]: RNase H (Rnase_H PF00075 RNase_HI_RT_DIRS1 cd09275, RNase_HI_RT_Ty1 cd09272, RNase_HI_RT_Ty3 cd09274), reverse transcriptase (RVT_2 PF07727, RVT_1 PF00078, RT_LTR cd01647, RT_DIRS1 cd03714), YR integrase (Phage_integrase PF00589, INT_Cre cd00799) and methyltransferase (Dam PF05869). Multiple sequence alignments of identified protein domains (YR, RNase H, RT) were prepared using PCMA [25]. Sequence conservation was visualized with WebLogo [26].

### CLANS clustering

Protein sequences of detected RT, RNase H, YR and MT protein domains were extracted, concatenated and clustered in the 3D mode in CLANS [27] with a p-value threshold of 1e-06. The dataset was further clustered in 2D mode with the *cluster in 2D* option in CLANS. Both 2D and 3D clustering resulted in the formation of the same groups, the 2D option was used for image preparation.

### Phylogenetic analyses

Concatenated protein sequences of detected RT, RNase H, YR and MT protein domains were also clustered with CD-HIT at 60% sequence identity and word length of 4 to filter out highly similar variants of each element. This step resulted in a list of 464 sequences. Protein sequences of identified RT, RNase H and YR protein domains from the 464 YR retrotransposons, eleven reference sequence of YR retrotransposons from RepBase and two LTR retrotransposon sequences (*D. melanogaster* (GI:148533491) and *D* *. buzzatii* (GI:4539021)) were used as an outgroup. The aforementioned sequences were aligned with the localpair iterative algorithm implemented in Mafft [28]. As MT is present solely in DIRS elements, only RT, RH and YR were used for YR retrotransposon phylogenetic analyses. Conserved columns from each multiple sequence alignment were selected with *strict* option in TrimAl [29]. The resulting set of 313 columns was concatenated with an in house Python script. The most suitable model for phylogenetic analysis was selected with ProtTest [30]. According to the AIC criterion, an LG+I+G+F model with a weighted score of 0.99 was chosen. Maximum likelihood analysis was carried out with PhyML, local version [31] with the following settings: LG model of amino acid substitution, 4 categories in gamma model with the shape parameter estimated as 1.156. In order to assess statistical reliability of estimated phylogenies branch support was calculated using an approximate likelihood ratio test [32]. For presentation purposes phylogenetic analysis were also performed on a smaller dataset comprising of concatenated RT, RH and YR domains from 28 YR TEs together with the same reference RepBase records and two LTR retrotransposons (379 columns in alignment after TrimAl trimming). Trees were visualized in iTOL [33] and Archaeoropteryx [34].

## Results

### Data set and enrichment in taxonomic groups

YR retrotransposons were detected with RepeatModeler and RepeatMasker [18] in 42 of 177 analyzed fungal genomes. Both full-length elements and remnant copies were considered, as our goal was to show evolutionary tendencies over exploring the current abundance of functional YR retrotransposons. However, only elements retaining detectable sequence either of integrase, transposase, methyltransferase or RNase H were analyzed (for details of the protein domain detection see below). The presence of at least one protein domain was considered as a sign of recent activity. 2241 representatives have been identified, the majority of them being either short, truncated or incomplete, often without or with incomplete repeats but still carrying at least a fragment of the aforementioned protein domains (genomic coordinates for all identified YR retroelements are provided in File S2).

**Figure 2:**
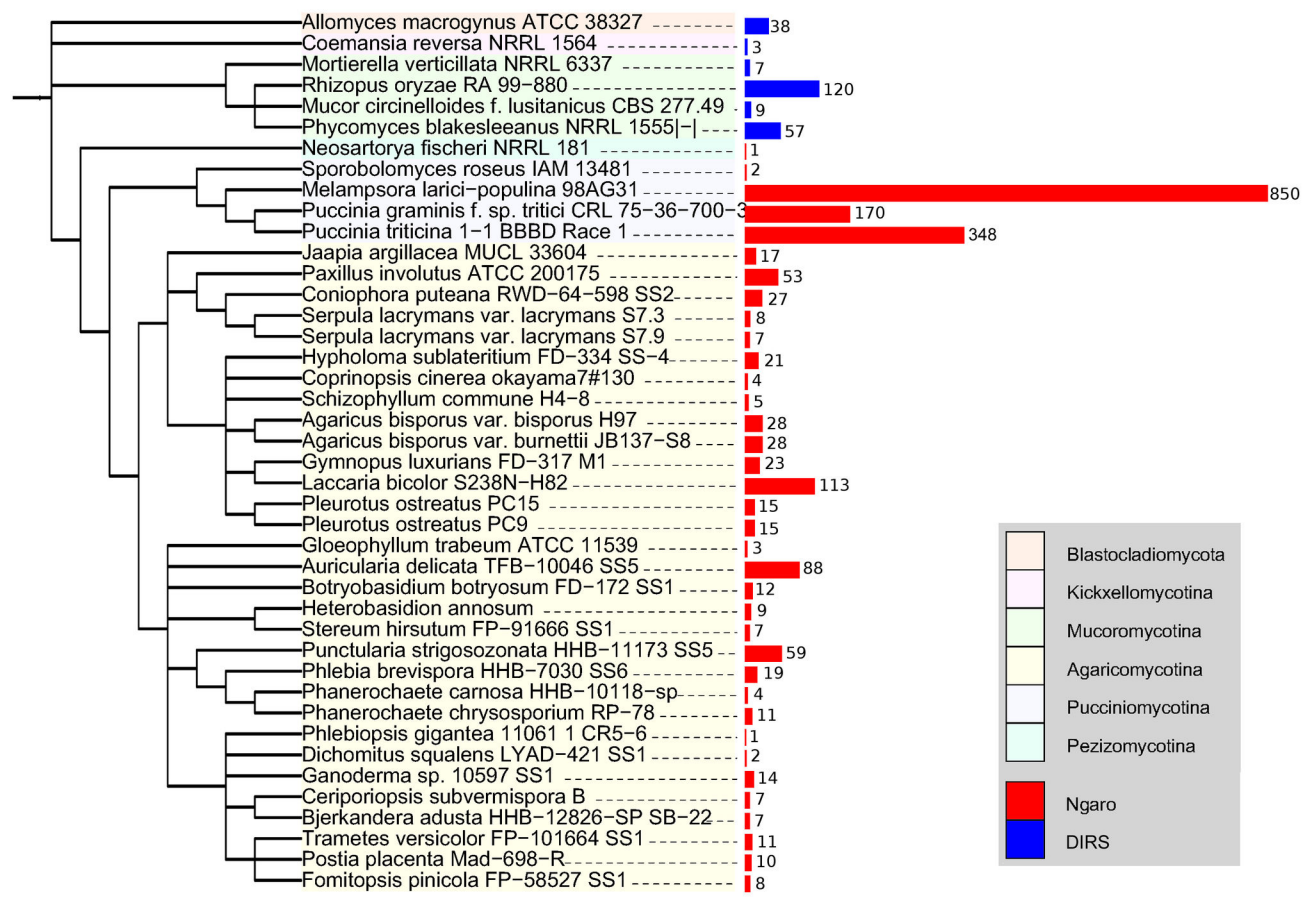
The number of YR elements per genome in 42 fungal genomes. The length of the bar is proportional to the number of detected DIRS and Ngaro retrotransposons. The schematic tree image was prepared with iTol [33].

The number of detected YR elements *per* genome is shown in Figure 2 (see File S3 for additional information on the number of remnant YR retrotransposons, genome assembly size, and the taxonomic classification of the organism). When comparing the total number of mobile elements between various genomes one should consider different genome sequencing methods and assembly algorithms, which may have strong impact on repetitive regions and can lead to the underrepresentation of mobile elements in the genome assembly. The taxonomic distribution of DIRS and Ngaro elements is almost disjoint. Fungal DIRS elements have been previously described from *Mucoromycotina* and Blastocladiomycota, exclusively [15]. Our results corroborate previous findings limiting DIRS distribution to early branching fungal lineages [14]. We found that *Allomycesmacrogynus* , representing Blastocladiomycota, has a genome abundant in YR elements (DIRS), while other basal fungi, *Batrachochytriumdendrobatidis* and *Spizellomyces* *punctatus* classified to Chytrydiomycota- lack detectable YR TE. Moreover, YR TEs were identified in all 4 sequenced *Mucoromycotina* representatives (*Mucor circinelloides*, *Mortierella* *verticillata* , *Rhizopus oryzae* and *Phycomycesblakesleeanus* ) and a single Kickxellomycotina ( *Coemansia* *reversa* ) genome. On the other hand, Ngaro elements were not found in genomes of any of the early branching fungi. They are Dikarya-specific, however only some of Basidiomycota genomes harbour these YR retrotransposons. Ngaro elements were detected in Pucciniomycotina and Agaricomycotina genomes but are absent from Ustilaginomycetes and Tremellomycetes. Three Pucciniomycotina genomes, known for their elevated genome size, contain more Ngaro elements than any other analyzed fungal genome. Agaricomycotina genomes in turn vary in the number of encoded Ngaro, but never reach the number of elements present in Pucciniomycotina genomes. Surprisingly, a single truncated Ngaro element was also identified in *Neosartorya* *fischeri* genome (supercontig 581:1068724-1071019) encoding RT, RH and YR domains, but without terminal repeats. However, the element is located within a cluster of mobile elements and both upstream and downstream regions have high sequence similarity to sequences from other Aspergillus taxa.

We wanted to determine both the number of elements per genome and the approximate abundance of families of YR elements in each genome. The latter may be assessed by clustering of encoded protein domains at 60% identity threshold. The obtained set of clustered sequences was subsequently used as representative for phylogenetic analyses. Our results show that genomes with higher number of identified elements (Pucciniomycotina) also have more families of YR elements (Fig. S1). This pattern is a likely consequence of recent amplification of mobile elements with simultaneous diversification of element families.

### Encoded protein domains

Evolutionary analysis and classification of retrotransposon are usually based on the presence of specific protein domains within their sequences. Following this approach, we detected typical YR retrotransposon protein domains: RT, RH, YR, and MT (only in DIRS) within identified transposable elements using HMMer and RPS-BLAST searches. The reverse transcriptase domain in Pfam database is represented by two separate protein family profiles: RVT_1 (PF00078) that includes retroviral and Ty3/Gypsy LTR retrotransposon sequences from all kingdoms of life, and RVT_2 (PF07727) encompassing predominantly Ty1/Copia LTR retrotransposons. The Pfam RNase H profile (PF00075) is built mostly on retroviral sequences which are known to differ from retrotransposon RNases [35]. The CDD database provides DIRS-directed RT_DIRS1 (cd03714) and RNase_HI_RT_DIRS1 (cd09275) profiles. Sequence searches performed with RT and RH domains from Pfam and CDD resulted in identification of twice as many RH domains comparing to RT sequences. Since RT and RH are co-occurring domains, they should appear together in the retroelement. A sequence profile built on representative sequences (obtained from clustering of DIRS and Ngaro RT sequences at 60% identity threshold) revealed that indeed, there are as many RT as RH domains in our YR retroelements collection (the reverse transcriptase HMM profile is available as File S4). The tyrosine integrase is described by a single protein family both in Pfam and CDD databases: Phage_integrase (PF00589) and INT_Cre (cd00799). Both YR domain profiles detect DIRS and Ngaro YR domains. DIRS DNA N-6-adenine-methyltransferase MT can be detected using Dam (PF05869) profile from Pfam database.

Importantly, based on protein domain composition and sequence repeats order we found that most retrotransposons described in RepBase as DIRS actually should be classified as Ngaro elements.

### Clustering and classification

**Figure 3:**
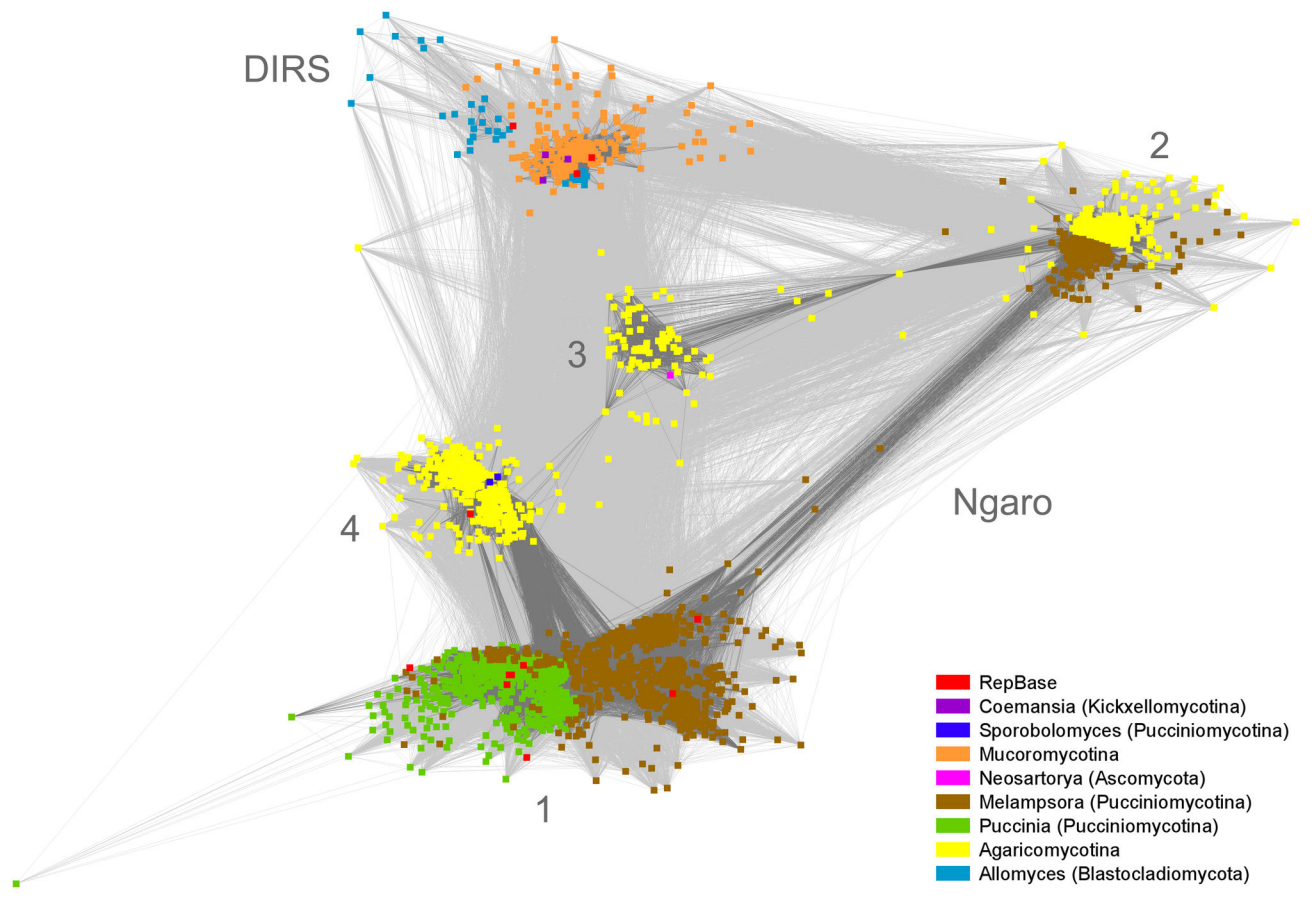
CLANS clustering of concatenated RT, RH, YR and MT domain sequences. The clustering was performed on the whole set of 2241 elements together with RepBase references. Ngaro clades are numbered. Connections with p-value <= 1e-40 are represented by dark grey lines and those with 1e-15 > p-value > 1e-40 are shown as light grey lines. A p-value threshold of 1e-06 was used for clustering.

Protein sequences corresponding to RT, YR, RH and MT protein domains from each identified YR element were concatenated and clustered together with reference DIRS and Ngaro sequences from RepBase using CLANS [27]. This step provided a graphical overview of major categories of YR retrotransposons. The clustering image clearly shows the separation of DIRS and Ngaro elements (Figure 3). All nodes in the graph form one connected component, no singletons were identified. All Ngaro groups contain representatives embracing all three protein domains (RT, RH, YR). Additionally, the clustering revealed the presence of four major Ngaro groups, of which only two include consensus profiles from RepBase. The new groups of Ngaro are well connected with known Ngaro elements (Figure 3, numbers 2 and 3). Consistently, all four Ngaro groups display strong pairwise similarities, which is reflected by their cycle-shaped arrangement (1-2-3-4) in CLANS clustering image. Elements located in-between clusters are composite retrotransposons with fragments from both neighboring clusters.

The first Ngaro group consists of Pucciniomycota sequences from three genomes: *M* *. laricis-populina* , *P* *. graminis* and *P* *. tritici* . The second group encompasses *M* *. laricis-populina* and sequences from all analysed Agaricomycotina families. The third group is Agaricomycotina-specific with a loss in Boletales ( *Paxillusinvolutus* , *Coniophoraputeana* , *Serpula* *lacrymans* ), Corticiales ( *Punctulariastrigosozonata* , *Phlebia* *brevispora* , *Phanerochaete* *carnosa* , *Phanerochaete chrysosporium*), *Gloeophyllumtrabeum* (Gloeophyllales) and *Botryobasidiumbotryosum* (Cantharellales), but present in other Agaricomycetidae (for example *Laccaria* *laccata* and *Pleurotus ostreatus*). The fourth group contains sequences from all Agaricomycotina families clustered together with the only RepBase representative CcNgaro3.

Agaricomycotina retain up to 3 different Ngaro groups in their genomes, whereas *M* *. larici-populina* (Pucciniomycotina) has 2 groups, and both Puccinia species have only one Ngaro group. Surprisingly, *Sporobolomyces* *roseus* , which is also a Pucciniomycotina representative, has two Agaricomycotina-type Ngaro elements, but no Ngaro elements similar to those found within Pucciniomycotina. Noteworthy, *S. roseus* has a much more compact genome than all 3 analyzed Pucciniomycotina.

### Phylogenetic analyses

In order to provide consistent classification of Ngaro elements and gain a better insight into YR retroelement evolution we performed phylogenetic analyses of representative elements. Concatenated protein sequences (RT, RH and YR) were clustered with CD-HIT to filter out similar variants of each element. Additionally, we added repeat sequences from RepBase as a reference and two LTR retrotransposons as outgroup sequences to the dataset. Each protein domain was aligned separately and the resulting alignments were consecutively concatenated and trimmed. The LTR retrotransposon sequences from *Drosophila* *buzzatii* (GI:4539021) and *D. melanogaster* (GI:148533491) were used to root the tree. These LTR retrotransposons encode RT and RH domains homologous to those from YR elements and have been successfully used in previous YR retrotransposon studies as an out-group for phylogenetic analyses [14].

**Figure 4:**
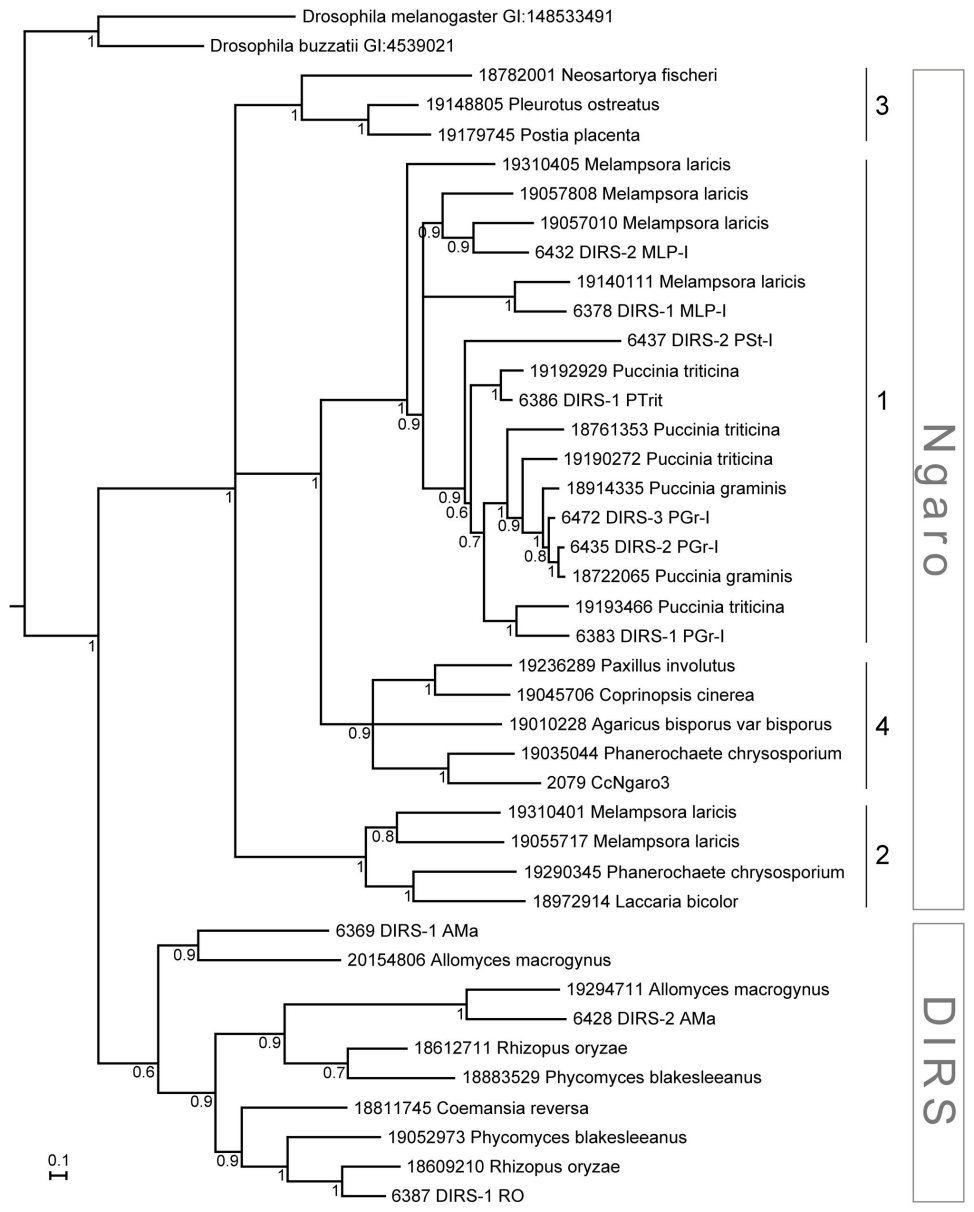
Maximum-likelihood phylogenetic tree of representative YR retrotransposons in analyzed fungal genomes. The phylogenetic analysis was performed with PhyML using concatenated amino acid sequences of RT, RH and YR protein domains. Approximate likelihood ratio test SH-like branch supports above 50% are shown. The tree image was prepared with Archaeopteryx [34].

DIRS and Ngaro elements are well separated on the obtained phylogenetic tree. There are two separate lineages clearly discernible among DIRS elements (Figure 4, Figure S1). One consists of *A* *. macrogynus* (Blastocladiomycota) sequences along with DIRS-1_AMa reference sequence and the latter includes *Mucoromycotina* ( *M* *. circinelloides* , *M* *. verticillata* , *R. oryzae* and *P* *. blakesleeanus* ) and Kickxellomycotina ( *C* *. reversa* ) sequences. The Blastocladiomycota / “Zygomycota” clade can be further divided into two groups, one of them labeled by the DIRS-1_RO from RepBase and the second by DIRS-2_AMa. Importantly, these clades display statistically significant support values. The topology of the DIRS subtree is consistent with sequence clustering results discussed above and with literature data. Some DIRS elements are shared by all “Zygomycota” genomes suggesting their common ancestry.

The Ngaro subtree is also statistically consistent and forms a trifurcation of Ngaro groups 1 & 4, 2 and 3. Each of the three branches displays reliable support values. The 1 & 4 clade forms a bifurcation leading to Agaricomycotina clade referred as Ngaro 4, and Pucciniomycotina clade labeled Ngaro 1 (Figure 3).

Ngaro 1 clade is composed of *M* *. larici-populina* and Puccinia sequences and contains multiple reference sequences: DIRS-2_PSt-I, DIRS-1_PTrit, DIRS-1_PGr-I, DIRS-2_PGr-I, DIRS-3_PGr-I, DIRS-1_MLP-I and DIRS-2_MLP-I. The reference sequences are widely distributed on the Ngaro 1 subtree showing a good coverage of the sequence diversity. *M* *. larici-populina* and Puccinia specific branches are clearly separated pointing at recent genome specific expansions of similar groups of elements (each sequence of the Figure S1 tree is a family representative).

Ngaro 4 elements seem to have mixed evolutionary history being inherited by many Agaricomycetes and further undergoing lineage specific expansions in some taxa, for example *Paxillusinvolutus* and *Punctulariastrigosozonata* .

Ngaro 2 clade has a clear bifurcation leading to a *M* *. larici-populina* and Agricomycotina branch and an Agaricomycotina specific branch. There are no reference sequences in either of the ramifications.

Ngaro 3 clade is composed of a single early branching *Neosartorya* *fischeri* sequence and a well separated Agaricomycotina-only clade. This is the smallest clade limited only to a handful of taxa with expansions of both element families and elements in *Pleurotus ostreatus* and *Auricularia* *delicata* . There are no reference elements representing this group in RepBase.

### Ngaro characterization

Retrotransposon sequences with potentially active RT, RH and YR protein domains were present in all four Ngaro clades. YR ORF can be either in the same frame as the RT-RH ORF or frameshifted in all Ngaro groups. On the nucleotide level only coding sequences can be reliably aligned within one cluster, what suggests that these elements have diversified a while ago. BLAST searches with a protein alignment of each of the 3 protein domains of each clade against the non-redundant protein database show some similarity to Ngaro elements from Metazoa (such examples can be found with E-value <= 1e-5). However, Ngaro clade 2 and Ngaro clade 3 alignments used as a query find more Metazoa sequences already in the top ranking hits with scores more significant that E-value <= 1e-20 suggesting they are related closer to animal Ngaro sequences than to Ngaro clade 1 and 4. This observation should be verified by further studies aimed at evolution of YR retotransposons in Eukaryota. In order to facilitate further Ngaro classification we analyzed protein motifs for each of the RT, RH and YR protein domains (Figure S2). Despite sharing common motifs that for example build respective active sites, the domains found in each clade retain their own characteristic sequence motifs that could be potentially used as descriptors when assigning new retrotransposon sequence to particular Ngaro group. Moreover, thorough analysis of sequence motifs shown significant similarity between Ngaro clades 1 and 4. Our results clearly demonstrate that in contrast to Ngaro clades 2 and 3, Ngaro clades 1 and 4 share unique motifs within all three protein domains.

## Discussion

### Taxonomic distribution

The most parsimonious scenario resulting in the present taxonomic distribution of YR retrotransposons in fungi comprises of at least two independent acquisitions of DIRS and Ngaro elements at two distinct time points. The presence of Ngaro almost exclusively in Basidiomycota and DIRS in early branching fungi may suggest that YR retrotransposons have evolved independently in Fungi.

Considering the polyphyletic origin of “Zygomycota” [36,37] and the presence of DIRS in *Mucoromycotina* , Kickxellomycotina and Blastocladiomycota, one might assume a common origin of all fungal DIRS elements with a loss in Dikarya. The primary acquisition might have occurred prior to Blastocladiomycota divergence. However, the presence of DIRS elements in Metazoa genomes supports their ancient origin going back to the Opisthokonta common ancestor. The most plausible hypothesis is a vertical inheritance of DIRS with subsequent diversification in both Metazoa and Fungi. On the other hand, we detected no YR retrotransposons in the *B* *. dendrobatidis* (Rhizophydiales) and *S* *. punctatus* (Spizellomycetales) genomes. The absence of YR retrotransposons in these genomes might have arisen due to: (i) species specific YR retrotransposon elimination, (ii) lineage specific YR retrotransposon loss in chytrids or (iii) acquisition of YR retrotransposons later in the course of fungal evolution. The latter is unlikely due to earlier Blastocladiomycota divergence.

Ngaro retrotransposons seem to have (i) either appeared in the common ancestor of Agaricomycotina and Pucciniomycotina after Ustilagomycotina divergence *via* horizontal gene transfer from an unknown Metazoa source, (ii) or invaded the Basidiomycota ancestor’s genome and consecutively disappeared from Ustilagomycotina genomes (or from the currently sequenced taxa), (iii) or have been acquired in more than one transfer leading to Metazoa-like Ngaro lineages (groups 2 and 3) and less similar to Metazoa Ngaro lineages (groups 1 and 4), (iv) or have been inherited from the common ancestor of Ophistokonta with loss in all fungal lineages but Agaricomycotina and Pucciniomycotina. Regardless of the way of transmission of ancestral Ngaro elements they seem to have been inherited and lost by many lineages. Some Ngaro subfamilies seem to have diverged in the ancestor of Agaricomycotina (Ngaro groups 3 and 4) and in the ancestor of Pucciniomycotina (Ngaro group 1). Only Ngaro group 2 is shared by a single Pucciniomycotina species and a number of Agaricomycotina representatives. This taxonomic puzzle is further complicated by the sequence similarity of Ngaro 2 and 3 to Metazoa Ngaro elements.

However, the presence of a Ngaro in a single Ascomycota ( *Neosartorya* *fischeri* ) seems to be an exception of unknown evolutionary provenance. The element is located within a cluster of mobile elements, a genomic region prone to retain fragments acquired *via* HGT. One might hypothesize about horizontal gene transfer occurring after speciation of the *A. fumigatus* group, but the possible donor is unknown. The position of *N* *. fischeri* sequence within the Ngaro clade 3, yet without high sequence similarity to other clade members gives no clues to this evolutionary puzzle.

### Ngaro classification

CLANS clustering and phylogenetic analyses indicate that four groups of Ngaro elements can be distinguished. These groups differ both in sequence and taxonomic distribution. The first one groups Pucciniomycota sequences from three genomes: *M* *. laricis-populina* , *P* *. graminis* and *P* *. tritici* . There are many subfamilies of elements in this group specific to each of the analyzed taxa and pointing at differentiation of group 1 elements after Pucciniomycotina radiation. The second group encompasses *M* *. laricis-populina* and sequences from all analyzed Agaricomycotina families. This taxonomic distribution is unexpected and might be a consequence of either a transfer to *M* *. laricis-populina* or ancestral inheritance with subsequent loss in other Pucciniomycotina representatives. The first hypothesis is supported by high sequence similarity within the group and good branch support values of the clade in the phylogenetic tree. The taxonomic distribution of the third group is unusual. These elements are present in some Agaricomycotina orders (Russullales, Polyporales, Auriculariales and Agaricales), but absent from other Agaricomycetidae: Boletales, Corticiales, Gloeophyllales and Cantharellales. The fourth group includes sequences from all of the analyzed Agaricomycotina.

RepBase reference sequences are available for two of the four groups. The first group is evenly sampled with reference sequences, while there is only a single reference sequence for the fourth clade. The development of consensus sequences for the remaining Ngaro groups will facilitate future genome annotation projects as the RepBase reference, together with RepeatMasker, are the tools of choice for mobile element annotation.

### Transposon-specific sequence profiles

Development of more specific protein domain profiles tuned towards mobile sequences is the indispensable step for further advancements in the field. Sequence profiles built on retroviral sequences are not sufficient for detection of protein domains in eukaryotic LTR retrotransposons [38] and YR retrotransposons (HMMer searches; E-value threshold of 0.1 for both RT and RH domains). Splitting the protein family into subfamilies and building sequence profiles specific either for protein type (retroviral *pol*, retrotransposon *pol*) or taxonomic distribution (prokaryotic/eukaryotic) in the way CDD does, seems to be a good direction. However, multiplying the number of protein family profiles into dozens is not within the scope of general protein domain databases such as Pfam and CDD. On the other hand, GypsyDB (http://gydb.org/index.php/Collection_HMM) is a resource devoted to retrotransposons [39]. GypsyDB stores a wide collection of sequence profiles for all protein domains present in known LTR and non-LTR retrotransposon families. These very narrow sequence profiles might be efficiently used not only for general protein domain annotation but preferentially for high resolution mobile element classification. Concluding, separate sequence profiles derived from non-model taxa and repetitive elements should improve protein domain detection within mobile elements.

### YR retroelements expansion

Most families and family expansions are genome-specific and might have emerged as a consequence of environmental conditions favoring genome variability. Our analysis reveals that 3 Pucciniomycotina taxa encode an elevated number of Ngaro elements. These genomes are known for their overall mobile element abundance and big genome size. The contribution of mobile elements to genome size growth has been discussed previously by Lynch [40,41]. Our findings seem to fit well into this model of overall mobile elements expansion. Similarly as observed for other groups of retrotransposons, Ngaro expansions seem to appear independently in distant taxa contributing to the increased genome size. Many of the identified Ngaro retrotransposons from Pucciniomycota retain a full set of protein domains what might be a hallmark of recent mobility. It is not clear what phenomena impacted the aforementioned taxa so they experienced a similar evolutionary scenario of independent genome expansion [9].

## Supporting Information

Figure S1 **Maximum-likelihood phylogenetic tree of YR transposable elements in the analyzed genomes.** The phylogenetic analysis was performed with PhyML using concatenated amino acid sequences of RT, RH and YR protein domains in 477 retroelements. Approximate likelihood ratio test SH-like branch supports above 50% are shown. The tree image was prepared with iTol [33].
(TIF)

Figure S2 **Sequence motifs identified within RT, RH and YR domains in four Ngaro groups.** Sequence conservation was visualized using WebLogo. Sequence motifs common to all Ngaro groups are aligned.
(PDF)

File S1 **List of fungal genomes used in this study.**
(XLS)

File S2 **YR retrotransposons identified in this study.** Genomic coordinates of all 2241 YR retrotransposons identified in 42 fungal genomes are provided.
(XLS)

File S3 **Additional characteristics of 42 fungal genomes with YR retrotransposons.** This table provides information about the number of YR retrotransposons with detectable protein domains and the number of remnant elements identified in this study. For a broader context the taxonomic classification, assembly size and number of encoded genes (as estimated by sequencing consortia) are given for each genome.
(XLS)

File S4 **YR retrotransposon RT HMM profile.** A HMM profile of the YR retrotransposon reverse transcriptase domain was built based on fungal YR retrotransposon sequences.
(HMM)
