# Supplementary figures and images for: DIRS and Ngaro Retrotransposons in Fungi
Source: PLoS One. 2013 Sep 25;8(9):e76319. doi: 10.1371/journal.pone.0076319 (PMC3783388; doi:10.1371/journal.pone.0076319)

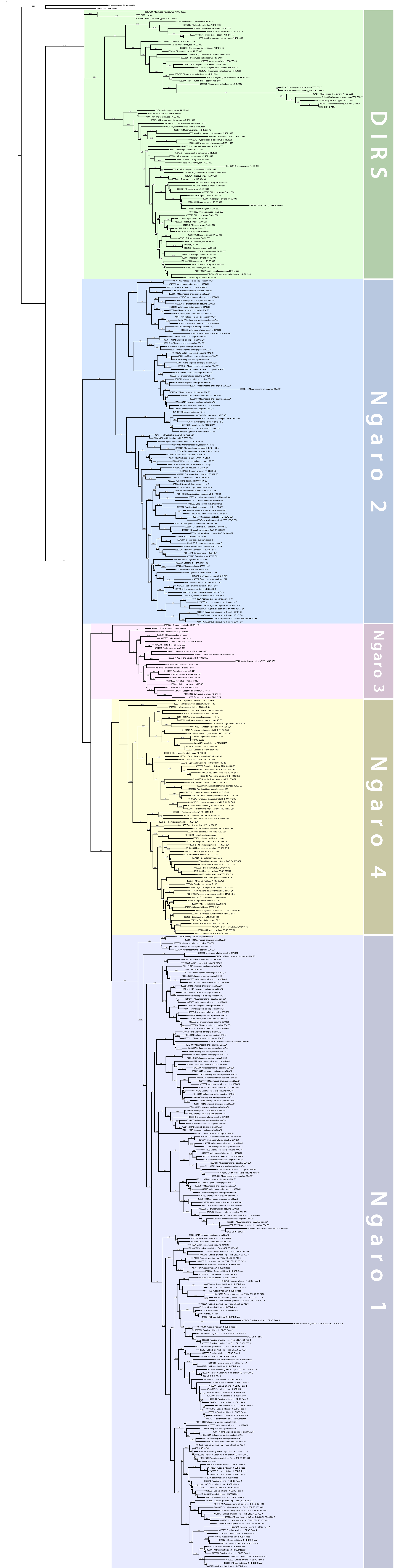

Supplement: Figure S1 — Maximum-likelihood phylogenetic tree of YR transposable elements in the analyzed genomes. The phylogenetic analysis was performed with PhyML using concatenated amino acid sequences of RT, RH and YR protein domains in 477 retroelements. Approximate likelihood ratio test SH-like branch supports above 50% are shown. The tree image was prepared with iTol [33]. (TIF) [file pone.0076319.s001.tif]

Reverse transcriptase

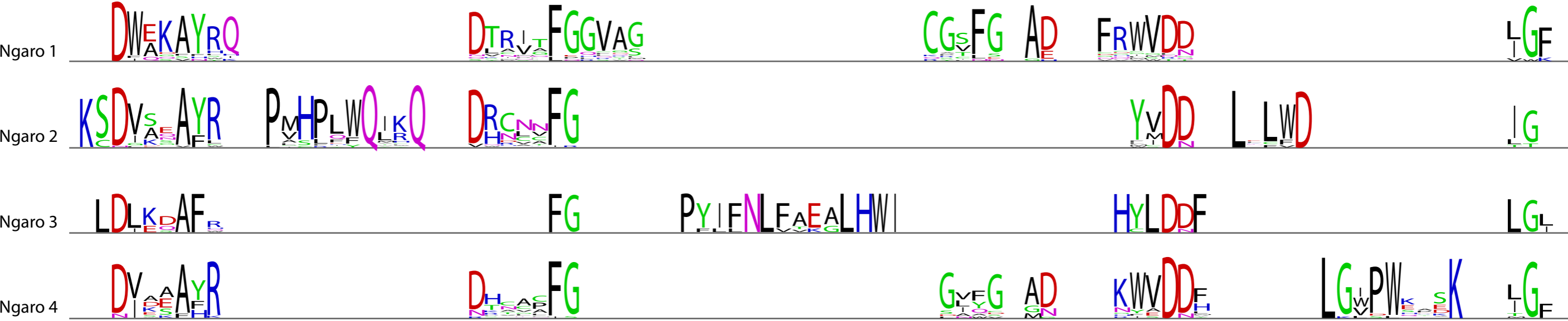

RNaseH

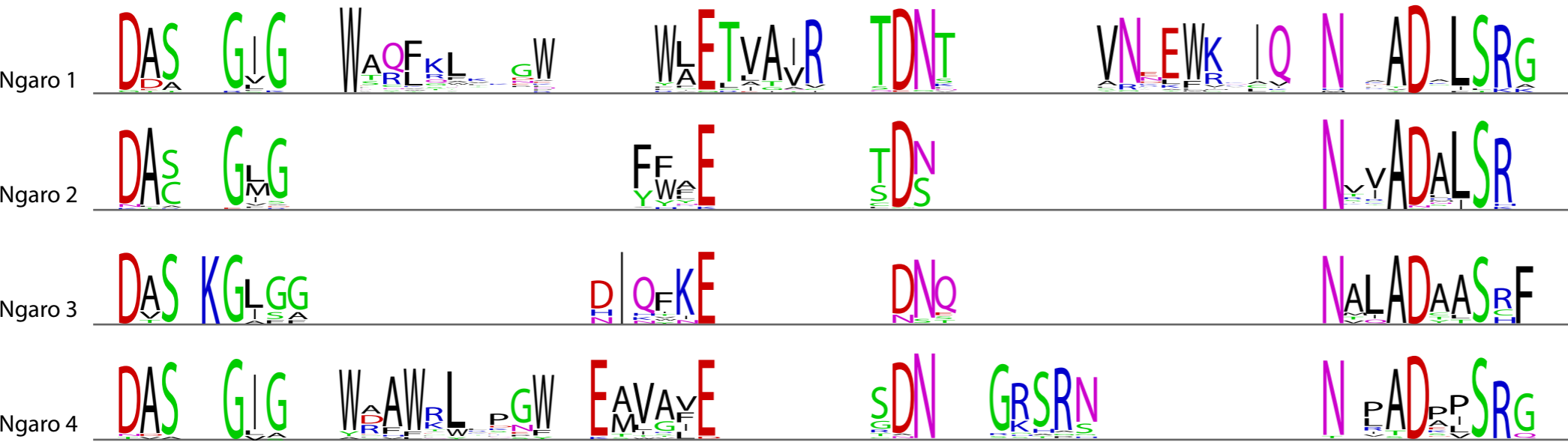

YR integrase

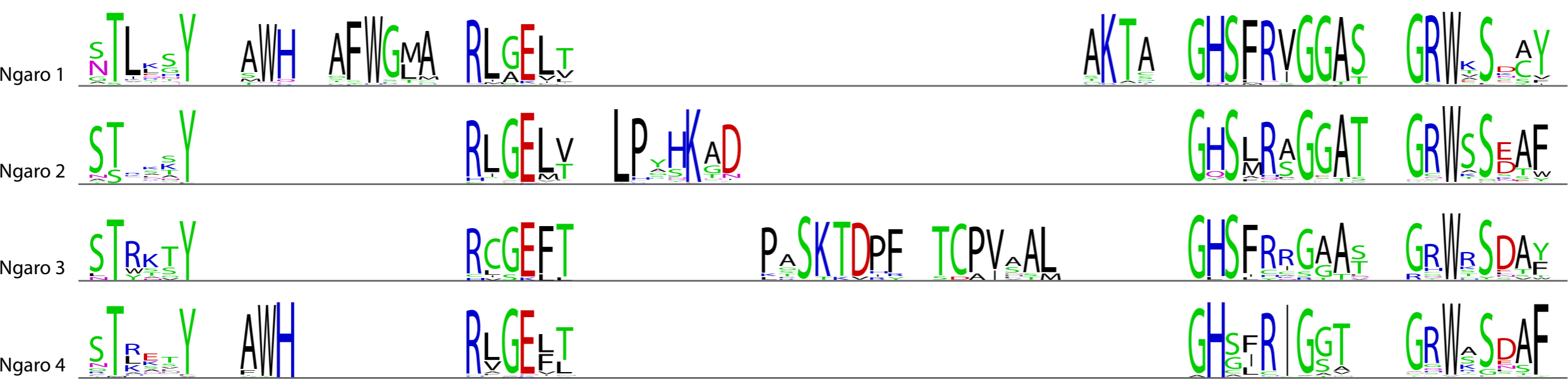

Supplement: Figure S2 — Sequence motifs identified within RT, RH and YR domains in four Ngaro groups. Sequence conservation was visualized using WebLogo. Sequence motifs common to all Ngaro groups are aligned. (PDF) [file pone.0076319.s002.pdf]
